# Synthesis, Structure, and Optoelectronic Properties of a Hybrid Organic–Inorganic Perovskite with a Monoethanolammonium Cation MA_x_MEA_1−x_PbI_3_

**DOI:** 10.3390/nano15070494

**Published:** 2025-03-26

**Authors:** Andrey Ryabko, Maxat Ovezov, Alexandr Tuchkovsky, Oleg Korepanov, Alexandr Maximov, Alexey Komolov, Eleonora Lazneva, Ekaterina Muratova, Igor Vrublevsky, Andrey Aleshin, Vyacheslav Moshnikov

**Affiliations:** 1 Laboratory of Nonequilibrium Processes in Semiconductors, Ioffe Institute, 26 Politekhnicheskaya, Saint Petersburg 194021, Russia; strontiumx94@gmail.com; 2 Department of Micro and Nanoelectronics, Belarusian State University of Informatics and Radioelectronics, 220013 Minsk, Belarus; a.tuchkovskij@bsuir.by (A.T.); vrublevsky@bsuir.edu.by (I.V.); 3 Department of Micro and Nanoelectronics, Saint Petersburg Electrotechnical University “LETI”, Saint Petersburg 197022, Russia; okrpnv@gmail.com (O.K.); aimaximov@mail.ru (A.M.); sokolovaeknik@yandex.ru (E.M.); vamoshnikov@mail.ru (V.M.); 4 Solid State Electronics Department, Saint Petersburg State University, Saint Petersburg 199034, Russiae.lazneva@spbu.ru (E.L.)

**Keywords:** perovskite, organic–inorganic, monoethanolammonium, cations, solar cell, crystal structure, UV–vis spectroscopy, morphology, hysteresis

## Abstract

Hybrid organic–inorganic perovskites have emerged as promising materials for next-generation optoelectronic devices owing to their tunable properties and low-cost fabrication. We report the synthesis of 3D hybrid perovskites with monoethanolammonium cations. Specifically, we investigated the optoelectronic properties and morphological characteristics of polycrystalline films of hybrid perovskites MA_x_MEA_1−x_PbI_3_, which contain methylammonium (MA) and monoethanolammonium (MEA) cations. MA_x_MEA_1−x_PbI_3_ crystallizes in a tetragonal perovskite structure. The substitution of methylammonium cations with monoethanolammonium ions led to an increase in the lattice parameters and the bandgap energy. Energy level diagrams of the synthesized samples were also constructed. The bandgap of MA_0.5_MEA_0.5_PbI_3_ makes it a promising material for use in tandem solar cells. These polycrystalline films, namely MA_0.5_MEA_0.5_PbI_3_ and MA_0.25_MEA_0.75_PbI_3_ were fabricated using a one-step spin-coating method without an antisolvent. These films exhibit a uniform surface morphology under the specified deposition parameters. Within the scope of this study, no evidence of dendritic structures or pinhole-type defects were observed. All synthesized samples demonstrated photocurrent generation under visible light illumination. Moreover, using monoethanolammonium cations reduced the hysteresis of the I–V characteristics, indicating improved device stability.

## 1. Introduction

The performance of hybrid organic–inorganic perovskite solar cells has led to a recent surge in interest in perovskite materials [1,2]. Inorganic and fully organic metal-free halogen perovskites have been the subject of more research due to the growing interest in hybrid perovskite materials [3,4,5,6,7,8]. Bulk (3D) hybrid perovskite materials (polycrystalline layers or single crystals), two-dimensional (2D), one-dimensional (1D), and zero-dimensional (0D) perovskite materials are thought to be very appealing for optoelectronic applications and have a wide range of uses [9,10,11,12,13,14,15,16,17].

A bandgap that is ideal for absorbing sunlight, a direct bandgap structure, a high absorption coefficient, high mobility, and a long average free path of the charge carriers are some of the special qualities that make hybrid perovskite solar cells effective [18,19]. By altering the composition of the hybrid perovskites, the bandgap can be adjusted, thereby controlling the absorption spectrum of the tandem solar cell. This is crucial for the growth of the silicon solar cell market, which may employ hybrid perovskites with a wider bandgap [20,21]. It is notable that a polycrystalline hybrid perovskite film can be produced from solution crystallized at low temperatures (about 100 °C). Perovskite films can be made using a variety of techniques, including spin coating, screen printing, inkjet, spraying, and doctor blades [2,22,23]. These application techniques have the advantage of being compatible with silicon solar cells and having low mass production costs. Hybrid perovskites can be utilized to make X-ray detectors, optically excited memristors, and light-emitting devices [24,25,26,27,28]. 

Currently, a wide range of compositions of polycrystalline films based on APbX_3_ has been proposed for the creation of photovoltaic structures, usually with a variation in the proportions of formamidinium (FA^+^, CH(NH_2_)_2_^+^) and methylammonium (MA^+^, CH_3_NH_3_^+^) cations and I^−^, Br^−^, and Cl^−^ anions, as well as the introduction of inorganic cations or partial replacement of lead [29,30,31,32]. The search for complex compositions of hybrid perovskites aims to improve stability while ensuring optimal values of the bandgap and solar cell efficiency [2]. Molecular passivation and the formation of films from 3D/2D perovskites can be used to reduce the degradation phenomena of perovskite photovoltaic structures [33,34,35,36]. For this purpose, as a rule, long-chain amines are used, which interact with uncoordinated Pb ions with terminal -NH_2_ groups and passivate defects at the periphery of grains. Also, some short-chain amines can act as organic linkers between inorganic octahedral frameworks in quasi-2D perovskites [34]. Nowadays, 2D perovskite organic–inorganic perovskite materials are divided into three phase types: Ruddlesden–Popper (RP) phase, Dion–Jacobson (DJ) phase, and alternating cations in interlayer space (ACI) phases and corresponding quasi-2D phases (layered perovskites) with general formulas A′_2_A_n−1_B_n_X_3n+1_ (for RP), A′A_n−1_B_n_X_3n+1_ (for DJ) и A′A_n_B_n_X_3n+1_ (for ACI), where n is the number of octahedral layers, A′ is spacer cations [9,37]. The chemical composition of 3D perovskites is typically designed so that the Goldschmidt tolerance factor falls within the range of 0.8 to 1.0. [37]. When the number of layers (n) is very large, quasi-2D layered perovskites resemble the 3D perovskite phase, typically with n much greater than 4. Adding cations of different sizes can stabilize the perovskite crystal structure and balance the Goldschmidt tolerance coefficient. A slight distortion of the lattice will occur when small amounts of larger cations are added [38]. Small amounts of certain cations that are utilized to create 2D and quasi-2D perovskites may also be added to the 3D perovskite crystal lattice [38,39,40].

Monoethanolammonium cation was used in [41] in 2004 to fabricate 2D perovskite (HO(CH_2_)_2_NH_3_)_2_PbX_4_, where (X = I, Br). A solar cell incorporating a 3D/2D heterojunction, composed of (HOOC(CH_2_)_4_NH_3_)_2_PbI_4_/CH_3_NH_3_PbI_3_ was fabricated. This device demonstrated exceptional operational stability under working conditions [42]. High permittivity (37.7) is another feature of the 2D perovskite (HO(CH_2_)_2_NH_3_)_2_PbX_4_. This is because the hydroxyl group and short carbon chain induce charge dipoles that greatly lower the exciton binding energy and increase the effectiveness of charge separation [43]. In [44], 3D hybrid perovskites based on monoethanolammonium and methylammonium cations were synthesized, characterized by a deficiency of lead and iodine compared to the MAPbI_3_ compound. Despite this, these materials retain a 3D architecture. The authors propose that such compounds can be regarded as an intermediate between 2D and 3D perovskites, which opens new possibilities for controlling their structural and functional properties. Moreover, the relatively high boiling point of ethanolamine (monoethanolamine) may contribute to its potential to lessen atmospheric perovskite degradation.

In this work, we investigated the effect of changing the proportion of monoethanolammonium cation MEA^+^ (HOCH_2_CH_2_NH^3+^) in 3D hybrid perovskite MA_x_MEA_1-x_PbI_3_. The influence of cation composition on the crystal lattice, optical properties, and energy level alignment was systematically examined. Additionally, changes in current–voltage characteristics and film morphology were analyzed. The polycrystalline layers were fabricated via one-step spin-coating from a DMF:DMSO (4:1) solution. In the composition range under investigation (up to a proportion of MEA = 0.75 with a proportion of MA = 0.25), the MA_x_MEA_1−x_PbI_3_ hybrid perovskites were found to be a stable perovskite phase. As the MEA fraction increases, both crystal lattice parameters and the bandgap increase with the shape changes of the absorption spectra. Our results demonstrate good agreement with the data presented in the study by A. Leblanc et al. [44]. In addition, perovskite phases are not formed by MEAPbI_3_. According to XRD and UV–VIS spectroscopy, MEAPbI_3_ is most likely in the δ-phase. Tuning the bandgap of MA_x_MEA_1−x_PbI_3_ makes it suitable for use in tandem solar cells. Comparing the current–voltage characteristics with those of MAPbI_3_, the hysteresis is reduced as the MEA fraction increases.

## 2. Materials and Methods

Monoethanolammonium iodide (MEAI, HOCH_2_CH_2_NH_3_I) was synthesized through the neutralization of monoethanolamine (HOCH_2_CH_2_NH_2_, JSC LenReactiv, St. Petersburg, Russia) with hydroiodic acid (HI, JSC LenReactiv, St. Petersburg, Russia) until a pH = 6.5 was attained. After that, the solution was evaporated at 90 °C in a water bath, and the precipitate that emerged was vacuum-filtered. MEAI and lead iodide (PbI_2_, Xi’an Yuri Solar Co., Ltd, Xi’an, China) were separately dissolved in dimethylformamide (DMF, C_3_H_7_NO, JSC LenReactiv, St. Petersburg, Russia). The two solutions were then mixed at a 1:1 molar ratio while maintaining a constant temperature of 60 °C throughout the process. Similarly, methylammonium iodide (MAI, CH_3_NH_3_I, Xi’an Yuri Solar Co., Ltd, Xi’an, China) and PbI_2_ were dissolved in DMF and combined in a 1:1 molar ratio at 60 °C to synthesize MAPbI_3_ (CH_3_-NH_3_PbI_3_).

By mixing the resultant MAI-PbI₂ and MEAI-PbI₂ solutions in 1:1, 3:1, and 1:3 ratios, precursor solutions were prepared for the synthesis of organic–inorganic perovskites with the general formula MA_x_MEA_1−x_PbI_3_ (MA_0.75_MEA_0.25_PbI_3_, MA_0.5_MEA_0.5_PbI_3_, and MA_0.25_MEA_0.75_PbI_3_).

These solutions were drop-casted onto glass substrates and subsequently annealed at 110–120 °C in an argon-inert atmosphere to facilitate crystallization and film formation. The resulting perovskite samples were then subjected to X-ray diffraction (XRD) analysis for structural characterization.

The thin layers of MA_x_MEA_1−x_PbI_3_ were deposited using a spin-coating technique with a solution of DMF and dimethyl sulfoxide (DMSO, C_2_H_6_OS, JSC LenReactiv, St. Petersburg, Russia) in a 4:1 volume ratio. DMSO increases the solubility limit of PbI_2_, and this DMF–DMSO ratio is commonly employed to facilitate the subsequent successful use of an antisolvent in the one-step spin-coating method for forming MAPbI_3_ films [45]. For MAPbI_3_ and MA_0.75_MEA_0.25_PbI_3_ films, an antisolvent was also utilized in the one-step spin-coating process. Ethyl acetate (EA, C_4_H_8_O_2_), which is less toxic compared to other suitable substances, was employed as the antisolvent [46]. The concentration of MA_x_MEA_1−x_PbI_3_ in the DMF and DMSO solution was approximately 400 mg/mL. The spin-coating process involved a centrifugation speed of 4000 rpm for 30 s, preceded by a preliminary centrifugation step at 1000 rpm for 10 s. When using the antisolvent, EA was applied during the centrifugation step at 4000 rpm. After centrifugation, the films were annealed for 10 min at 110 °C on a hotplate. Both the annealing and centrifugation processes were carried out in an inert argon (Ar) atmosphere within a glovebox. The films were deposited on glass substrates for spectrophotometric and morphological studies. For the evaluation of current–voltage characteristics (IVs), perovskite films were spin-coated onto ceramic substrates equipped with interdigitated gold electrodes (Sensor Platform, Tesla Blatna, a.s., Blatna, Czech Republic). The interdigitated electrodes consist of gold (Au) strips with a spacing of 25 μm between adjacent strips and a strip width of 25 μm. The total active area of the electrode array measures 4.2 × 4.2 mm.

For ultraviolet photoelectron spectroscopy (UPS), the MA_0.75_MEA_0.25_PbI_2_, MA_0.5_MEA_0.5_PbI_2,_ and MA_0.25_MEA_0.75_PbI_2_ samples were deposited on glass substrates with an ITO (Indium Tin Oxide) layer similarly to centrifugation, but using solutions with a concentration of ~40 mg/mL.

Photoelectron spectroscopy of the samples was carried out under ultrahigh vacuum conditions (~10^−7^ Pa) on an Escalab 250Xi complex photoelectron spectrometer (Thermo Fisher Scientific Inc., Waltham, MA, USA) with a photon excitation energy of hν(He I) ≈ 21.2 eV for UPS and of AlKα = 1486 eV for X-ray photoelectron spectroscopy (XPS). XPS spectra were processed using CasaXPS Version 2.3.24 software. Carbon-containing and oxygen-containing surface adsorbates might drastically distort the relative UPS peak positions as well as blur the whole UPS spectrum. Oxygen adatoms might be involved in the formation of metal oxide components and carbonyl functional groups [47]. To avoid such distortions, we used Ar^+^ sputtering accelerated by 500 V voltage for 20–30 s as we described in more detail in our previous work [48,49].

XRD measurements of the samples were carried out using a Bruker D2 PHASER X-ray diffractometer (Bruker, Billerica, MA, USA) in a 2θ angle range of 10 to 45° with a scanning rate of 1° per minute using CuKα radiation.

Absorption spectra were acquired using a SPECS SSP 715 UV–Vis spectrophotometer (Spectroscopic Systems, Moscow, Russia). The topography of the organic–inorganic perovskite films was investigated using an atomic force microscope (AFM) NTEGRA (NT-MDT, Moscow, Russia) and a POLAM-312 polarization microscope (LOMO, St. Petersburg, Russia).

IVs were measured using a Keithley 6487 picoammeter (Keithley Instruments, Solon, OH, USA). The measurements were conducted in the dark, with the applied voltage swept across a range of −2.5 to 2.5 V. To evaluate the photoresponse of the perovskite films, a green LED light source with a peak wavelength of approximately 535 nm and an irradiance of 100 W/m^2^ was employed. The films, deposited on interdigitated electrodes, were exposed to this light source during the photoresponse measurements.

## 3. Results and Discussion

**Figure 1:**
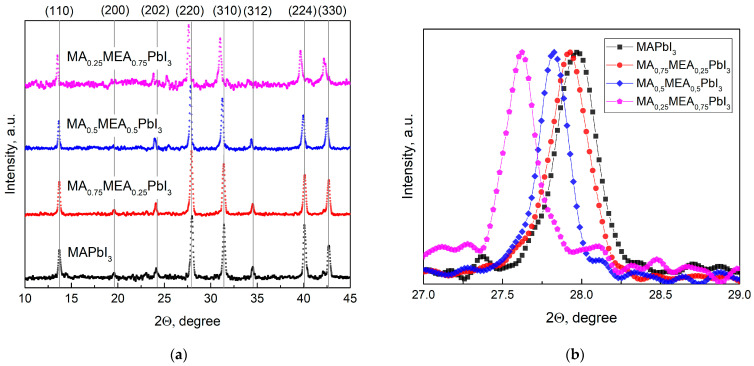
XRD patterns of obtained samples in (**a**) 10–45° range; (**b**) 27–29° range (planes (220)).

Figure 1a displays the XRD patterns for the perovskites MA_0.25_MEA_0.75_PbI_3_, MA_0.5_MEA_0.75_PbI_3_, MA_0.75_MEA_0.25_PbI_3,_ and MAPbI_3_. The obtained samples confirmed the tetragonal structure of the obtained perovskites with minor peaks of planes (200), (202), and (312), and intense peaks assigned to planes (110), (220), (310), (224), and (330) [44,50].

As the MEA fraction increases, XRD peaks shift to lower 2Θ angles, indicating expanded interplanar spacing. This correlates with a blue shift in the absorption edge and an increase in bandgap energy. This trend is clearly demonstrated in Figure 1b, which focuses on the (220) plane, the most intense peak in the samples. The systematic peak shift to lower 2Θ values with higher MEA concentration confirms the lattice expansion. These results suggest that the MA_x_MEA_1−x_PbI_3_ perovskites remain structurally stable across the studied composition range.

The data obtained in our study are in good agreement with the results presented in the work by Leblanc et al. [44]. In the aforementioned study, the crystal structure of the hybrid perovskite was thoroughly investigated using MA^+^ and MEA^+^ cations (in combination with Pb^2+^ and I^−^), confirming the formation of a three-dimensional (3D) hybrid perovskite with a tetragonal structure. It was demonstrated that an increase in the proportion of the MEA^+^ cation leads to a decrease in the 2Θ angles for the main peaks, indicating an expansion of interplanar distances. Notably, in our independent study, the goal was not to intentionally reduce the proportion of Pb^2+^ and I^−^ to obtain compounds of the composition (MA)_1−2.48x_(MEA)_3.48x_[Pb_1−x_I_3−x_]; however, the obtained results demonstrate good consistency with the data from [44].

To calculate the effective tolerance factor (t_eff_) for compositions of the type A_x_B_1−x_X_3_ (in our case, MA_x_MEA_1−x_PbI_3_), a previously proposed approach based on the calculation of the effective cation size (r_eff_) can be used [51]. Specifically, the calculation of t_eff_ for the perovskites MAPbI_3_, MA_0.75_MEA_0.25_PbI_3_, MA_0.5_MEA_0.5_PbI_3_, MA_0.25_MEA_0.75_PbI_3_, and MEAPbI_3_ was performed using the following formulas:

(1) $$r_{eff}={x\cdot r}_{{MA}^{+}}+(1-x)\cdot r_{{MEA}^{+}}$$

(2) $$t_{eff}=\frac{r_{eff}+r_{I^{-}}}{\sqrt{2}\cdot (r_{{Pb}^{2+}}+r_{I^{-}})}$$

The ionic radii were assumed to be 0.132 nm for Pb^2+^, 0.206 nm for I^−^, 0.18 nm for MA^+^, and 0.24 nm for MEA^+^ [52,53]. The calculated values of the effective tolerance factor (t_eff_) were 0.81, 0.84, 0.87, 0.90, and 0.93 for the perovskites MAPbI_3_, MA_0.75_MEA_0.25_PbI_3_, MA_0.5_MEA_0.5_PbI_3_, MA_0.25_MEA_0.75_PbI_3_, and MEAPbI_3_, respectively. This approach demonstrates that the size of the MEA^+^ cation may be suitable for the formation of a perovskite crystal structure, providing an effective tolerance factor within the range of 0.8 to 1. However, the use of MEA^+^ may lead to the formation of intermolecular hydrogen bonds due to interactions of the NH^3+^···OH type [41]. In our view, the likelihood of hydrogen bond formation between cations such as MEA^+^ and MA^+^, as well as between MEA^+^ and MEA^+^ cations, may contribute to the formation of a 3D hybrid perovskite with channels in the crystal structure (deficiency of Pb^2+^ and I^−^), which is consistent with the results described by Leblanc et al. [44].

**Figure 2:**
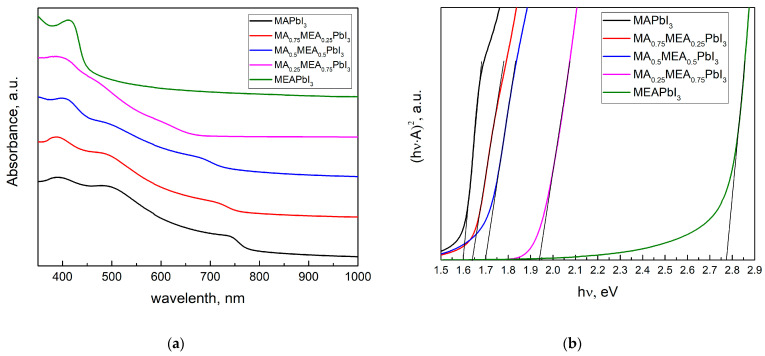
UV–vis spectra of MA_X_MEA_1−x_PbI_3_ thin films (**a**); the same spectra in Tauc plot (**b**).

Figure 2a displays the perovskite film’s absorption spectra. The absorption edge of MA_x_MEA_1−x_PbI_3_ shifts towards shorter wavelengths (blue shift) as the MEA fraction increases, indicating an increase in the bandgap energy (Figure 2b).

The optical bandgap E_g_ was determined in the Tauc coordinates (A·hν)^1/r^ from hν by extrapolating the linear section to the energy coordinate, where r is determined by the type of dependence of the absorption coefficient of the semiconductor on the irradiation energy greater than the bandgap. For direct-gap semiconductors, the absorption coefficient is described by the root dependence (r = 1/2) [54]. The absorption spectrum remains nearly unchanged (Figure 2a), and the bandgap energy does not significantly increase even when MEA cations constitute up to 25% of the total cation concentration, as shown by UV–vis spectra in Tauc plot in Figure 2b. Therefore, incorporating MEA cations in MA_x_MEA_1−x_PbI_3_ up to x = 0.25 does not significantly reduce the material’s absorption of solar radiation. For MA_0.25_MEA_0.75_PbI_3_, a notable increase in the bandgap E_g_ = 1.94 eV was observed. These data are consistent with the increase in the interplanar distances in the MA_x_MEA_1−x_PbI_3_ crystal lattices. The shift of peaks in MA_0.75_MEA_0.25_PbI_3_ and MA_0.5_MEA_0.5_PbI_3_ to the region of smaller 2Θ angles is less noticeable than the shift of peaks in MA_0.25_MEA_0.75_PbI_3_, as illustrated in Figure 1b. The interplanar spacing of the crystal lattice and the bandgap energy (E_g_) exhibit a nonlinear dependence on the MEA cation fraction in MA_x_MEA_1−x_PbI_3_. A similar sharp increase in the optical bandgap, observed for the perovskite MA_0.25_MEA_0.75_PbI_3_ (E_g_ ≈ 1.94 eV), was also recorded for the composition (MEA)_0.73_(MA)_0.47_[Pb_0.80_I_2.80_] (Eg ≈ 1.84 eV), which is structurally the closest. In this case, Pb^2+^ and I^−^ ions are absent in the channels of the crystal lattice, and the increase in the bandgap in the proposed model of the crystal structure is explained by the disruption of connectivity in the PbI_6_ octahedra along the crystallographic directions 2a + b and a − 2b [44]. This model of the crystal structure is of significant interest and can be considered a promising approach to explaining the observed phenomena. Overall, our results, demonstrating an increase in the bandgap with the expansion of interplanar distances in the 3D hybrid perovskite MA_x_MEA_1−x_PbI_3_, are consistent with previously obtained data and confirm their reliability.

Similar results regarding the formation of a 3D-vacant perovskite structure with a tetragonal crystal lattice were observed in the MEA_x_FA_1−x_SnI_3_ system for x = 0.6–1.0. For x = 0–0.2, an orthorhombic crystal structure was observed, while for x = 0.2–0.4, a rhombohedral structure was identified [55]. It can be hypothesized that in the MA_x_MEA_1−x_PbI_3_ system, there is a competition between the incorporation of the MEA^+^ cation into the crystal lattice and the formation of channels within the structure, depending on the proportion of MEA^+^ as well as the ratios of Pb^2+^ and I^−^. The significant increase in the bandgap from Eg ≈ 1.7 eV for MA_0.5_MEA_0.5_PbI_3_ to Eg ≈ 1.94 eV for MA_0.25_MEA_0.75_PbI_3_ may also be attributed to the formation of channels induced by the high proportion of MEA^+^. These hypotheses require further comprehensive studies, including both theoretical calculations and direct observation of channels in the crystal structure, which is beyond the scope of this work.

The value of the bandgap E_g_ ≈ 1.7 eV for MA_0.5_MEA_0.5_PbI_3_ is in the range of preferred E_g_ values for the perovskite film in the top photovoltaic structure in silicon-based tandem solar cells [56,57]. The bandgap energy of MA_x_MEA_1−x_PbI_3_ can be tuned from approximately 1.6 eV (for x = 1, pure MAPbI_3_) to 1.94 eV (for x = 0.25, MA_0.25_MEA_0.75_PbI_3_), making it possible to optimize the material for specific applications, such as tandem solar cells.

**Figure 3:**
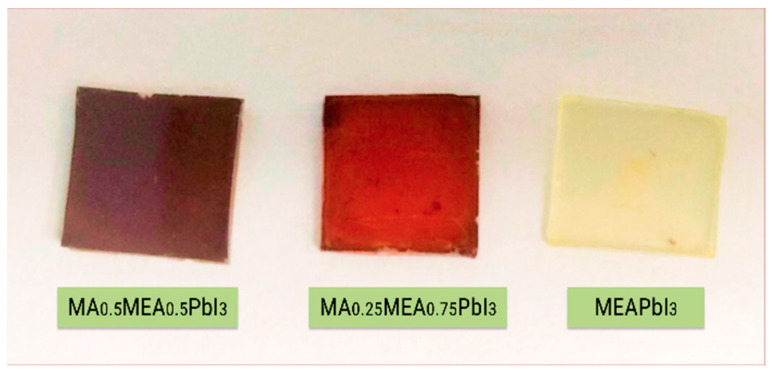
Photography of MA_x_MEA_1−x_PbI_3_ films and MEAPbI_3_ film on glass substrates.

**Figure 4:**
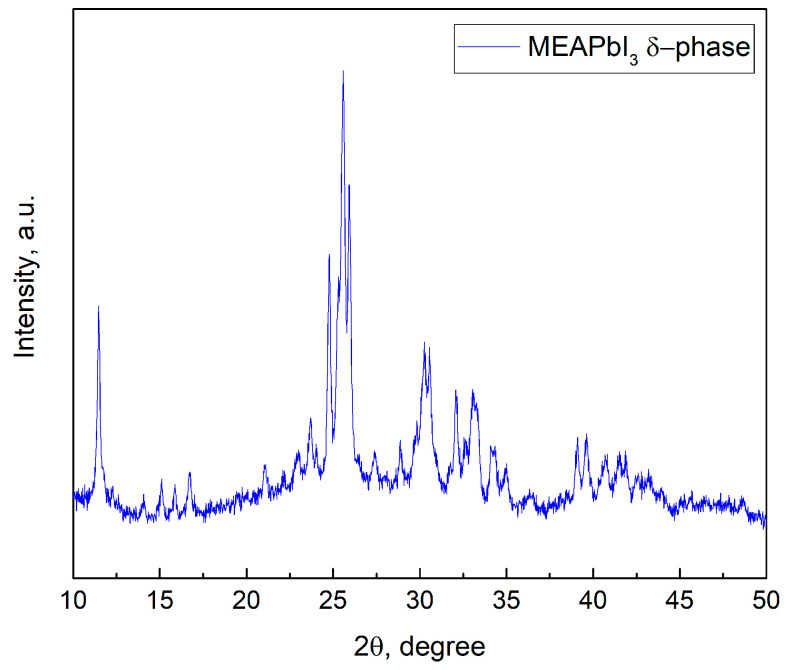
XRD pattern of MEAPbI_3_ film.

Analysis of the absorption spectra indicates a notable shift in the absorption edge of MEAPbI_3_ relative to the MA_x_MEA_1−x_PbI_3_ samples, with an optical bandgap of ~2.77 eV. This value exceeds the bandgap energies of both the two-dimensional perovskite MEA_2_PbI_4_ and PbI_2_ [41,58]. As depicted in Figure 3, the MEAPbI_3_ film displayed a distinct yellow coloration. Additionally, the XRD patterns (Figure 4) suggest the formation of a non-perovskite phase, indicating that MEAPbI_3_ does not crystallize into the conventional perovskite structure under these conditions.

The CsPbI_3_ samples without additives exhibited similar behavior, forming an orthorhombic (δ) phase with identical XRD peak positions and no perovskite crystal structure [59]. Under certain conditions, hybrid perovskites incorporating common cations such as MA and FA can also adopt the δ-phase [60,61]. Thus, despite the fact that the effective tolerance factor (t_eff_) for the MEAPbI_3_ compound is less than 1, the increased bandgap compared to PbI_2_, along with the XRD data, suggests the formation of the δ-phase in MEAPbI_3_. However, the primary conclusion of this study is that MEAPbI_3_ adopts a non-perovskite structure under the given experimental conditions.

**Figure 5:**
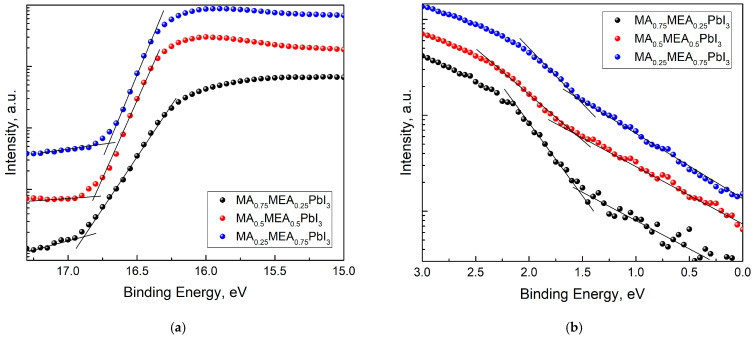
UPS spectra of MA_0.75_MEA_0.25_PbI_3_, MA_0.5_MEA_0.5_PbI_3_, MA_0.25_MEA_0.75_PbI_3_: (**a**) cutoff energy region UPS; (**b**) onset energy (VB edge) region UPS.

For MA_0.75_MEA_0.25_PbI_3_, MA_0.5_MEA_0.5_PbI_3_, and MA_0.25_MEA_0.75_PbI_3_ organic–inorganic perovskites, the cutoff values for the high binding energy (E_cutoff_) and the initial binding energy (E_onset_) were determined (Figure 5). With the irradiation energy of 21.21 eV subtracted from E_cutoff_, the work functions (Fermi level E_F_ relative to vacuum) for the perovskites were A(MA_0.75_MEA_0.25_PbI_3_) = 4.33 eV, A(MA_0.5_MEA_0.5_PbI_3_) = 4.40 eV, and A(MA_0.25_MEA_0.75_PbI_3_) = 4.51 eV, respectively. To determine the value of the valence band maximum (VBM) relative to vacuum, the value of the initial binding energy (E_onset_) was added to the work function A, that is, the following formula was used:

−VBM = hν − (E_cutoff_ − E_onset_). (3)

The VBM calculated values were 5.87 eV, 5.96 eV, and 6.07 eV for MA_0.75_MEA_0.25_PbI_3,_ MA_0.5_MEA_0.5_PbI_3_, and MA_0.25_MEA_0.75_PbI_3_, respectively.

**Figure 6:**
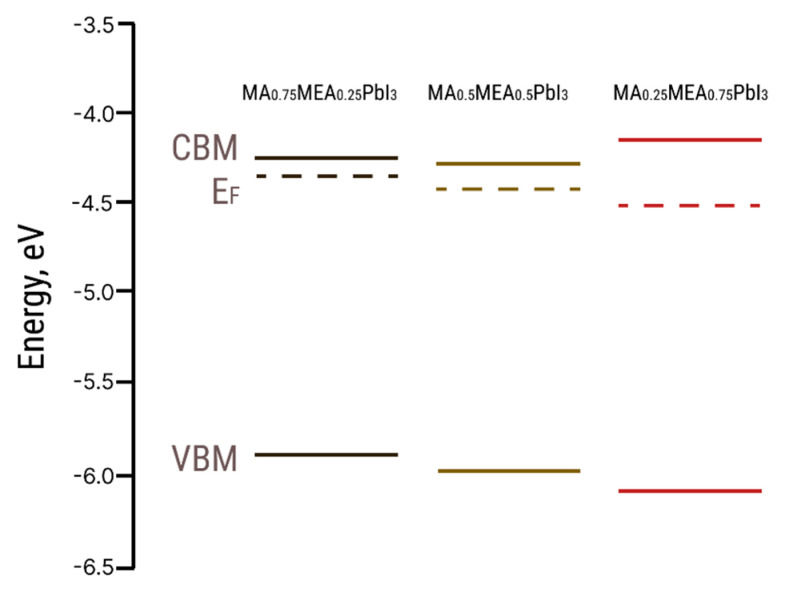
Energy level diagrams for MA_0.75_MEA_0.25_PbI_3_, MA_0.5_MEA_0.5_PbI_3,_ and MA_0.25_MEA_0.75_PbI_3_ hybrid perovskites (CBM—conduction band minimum, VBM—valence band maximum, E_F_—Fermi level).

The E_F_ values from UPS and the optical bandgap Eg values were used to construct the energy band diagram for MA_0.75_MEA_0.25_PbI_3_, MA_0.5_MEA_0.5_PbI_3_, and MA_0.25_MEA_0.75_PbI_3_ (Figure 6).

**Figure 7:**
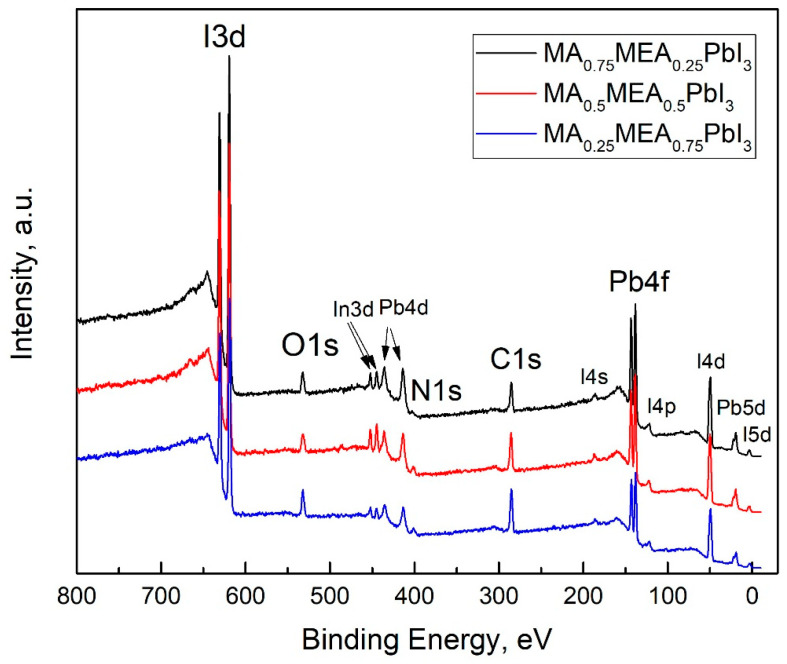
Survey XPS spectra of thin films of MA_0.75_MEA_0.25_PbI_3_, MA_0.5_MEA_0.5_PbI_3_, and MA_0.25_MEA_0.75_PbI_3_ deposited on ITO-coated substrates.

The VBM and E_F_ values for MA_0.75_MEA_0.25_PbI_3_ are very close to MAPbI_3_ [62,63]. This similarity may be attributed to the insignificant influence of the small fraction of MEA cations on the lattice structure, band structure, and crystal lattice parameters. The Fermi level E_F_ is also influenced by deviations in the perovskite composition from its stoichiometric ratio (i.e., the atomic ratio N:Pb:I). In the case of MA_0.75_MEA_0.25_PbI_3_, our measurements revealed a significant excess of Pb atoms relative to N and a slight deficiency of I atoms compared to Pb. Such deviations can induce n-type self-doping, likely due to the formation of electron-donating defects associated with Pb excess and I vacancies [64]. It is worth noting that the samples were briefly exposed to air prior to XPS analysis, which may have contributed to the partial evaporation of methylamine. For MA_0.5_MEA_0.5_PbI_3_, an excess of Pb was also detected, though it was less pronounced than in MA_0.75_MEA_0.25_PbI_3_. In contrast, the atomic ratio of N:Pb:I in MA_0.25_MEA_0.75_PbI_3_ was found to be closest to the stoichiometric ratio of 1:1:3. On the survey X-ray photoelectron spectroscopy (XPS) spectra presented in Figure 7, the most intense peaks corresponding to the core levels of I3d, Pb4f, N1s, C1s, and O1s are indicated. Additionally, less intense peaks are marked, with their identification carried out in accordance with the literature data [65,66,67].

It should also be noted that the UPS data of perovskite films are highly sensitive to experimental conditions, particularly the choice of substrate and film thickness. These factors can induce Fermi-level pinning, which may significantly affect the measured electronic properties [68]. The peaks of the In3d core level are associated with the ITO material at the grain boundaries of the thin films. Accordingly, the peak of the O1s core level is attributed not only to the presence of the OH group in the MEA^+^ cation but also to the oxygen of ITO, as well as the possible presence of adsorbed OH groups on the surface of the samples.

**Figure 8:**
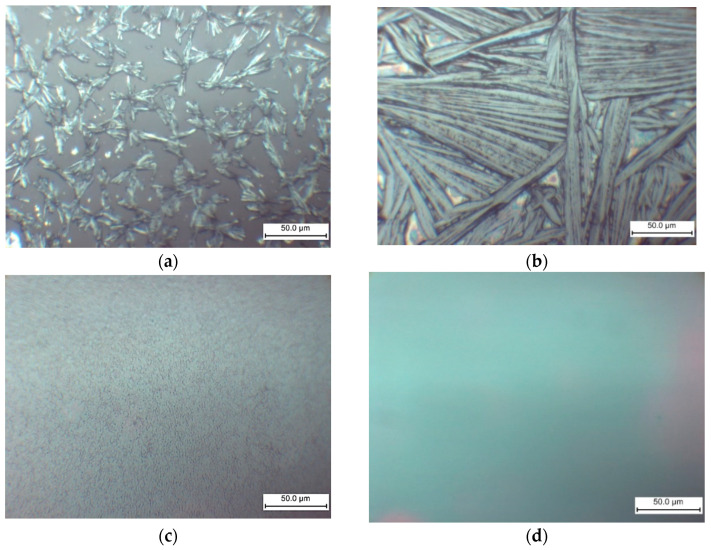
Optical microscopy images of MA_x_MEA_1−x_PbI_3_ films obtained by one-step centrifugation from a solution of DMF and DMSO (1:4) on glass substrates: (**a**) MAPbI_3_; (**b**) MA_0.75_MEA_0.25_PbI_3_; (**c**) MA_0.5_MEA_0.5_PbI_3_; (**d**) MA_0.25_MEA_0.75_PbI_3_.

The morphology of the films formed on the glass substrate is significantly influenced by the MEAI content in the solution, as demonstrated by the optical microscopy results (Figure 8). Therefore, it is anticipated that the MEAI concentration will have a significant impact under other film acquisition conditions.

When perovskite films are deposited without the use of an antisolvent, the MAPbI_3_ film forms as elongated split crystalline (or dendritic structures) with a length of about 25 μm (Figure 8a). The formation of MAPbI_3_ elongated split crystals may also be observed in DMF without the addition of DMSO [69,70]. During the crystallization of MAPbI_3_, homogeneous nucleation in the near-surface region of a thin film of the solution is associated with the formation of such structures in [69]. As shown in Figure 8, the addition of MEAI to the solution has a significant effect on the morphology of the film. The size of the elongated split perovskite crystallites thus increases sharply to a characteristic size of 0.1–0.2 mm at 25% MEAI and 75% MAI (Figure 8b). These structures’ extremely high relief makes them unsuitable for the production of solar cells, but they might be useful for building planar structures and for applications as X-ray or photodetectors. Increasing the MEAI fraction further produces a continuous homogenous coating and layers devoid of dendritic structures.

**Figure 9:**
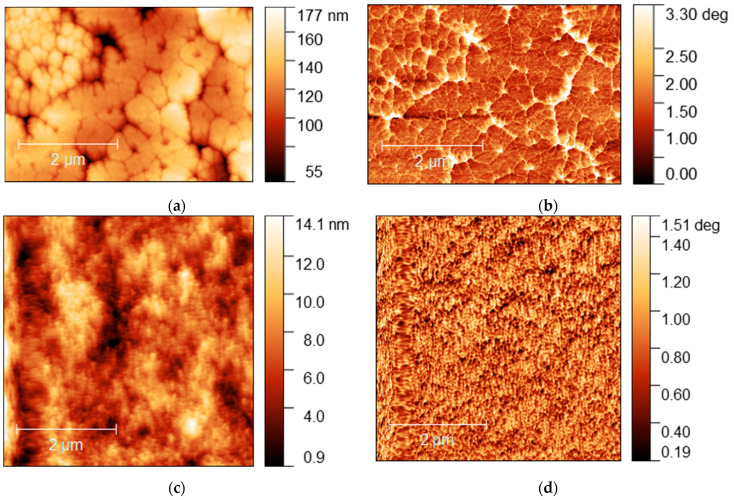
AFM images of MA_0.5_MEA_0.5_PbI_3_ film: (**a**) topography and (**b**) phase contrast; AFM image of MA_0.25_MEA_0.75_PbI_3_ film: (**c**) topography and (**d**) phase contrast.

A significant difference in morphology was also observed by AFM of samples MA_0.5_MEA_0.5_PbI_3_ and MA_0.25_MEA_0.75_PbI_3_ (Figure 9).

The MA_0.5_MEA_0.5_PbI_3_ film has a hierarchical structure, with larger grains (0.5–1 μm) made up of 50 nm nanocrystallites (Figure 9a,b). And the perovskite film MA_0.25_MEA_0.75_PbI_3_ is an even more uniform coating of 50 nm nanocrystallites (Figure 9c,d). Thus, a change in the concentration of MEAI obviously significantly affects the crystallization processes of perovskite films. The relief of the film MA_0.5_MEA_0.5_PbI_3_ is about 150 nm, while the relief of MA_0.25_MEA_0.75_PbI_3_ is around 15 nm (for an approximate film thickness of 400–500 nm). This leads to the fact that visually the surface of the film MA_0.25_MEA_0.75_PbI_3_ looks mirror-like. Thus, MA_0.5_MEA_0.5_PbI_3_ and MA_0.25_MEA_0.75_PbI_3_ films using DMF and DMSO (4:1) as a solvent are formed as continuous homogeneous coatings, which can be used to create perovskite solar cells without using an antisolvent.

**Figure 10:**
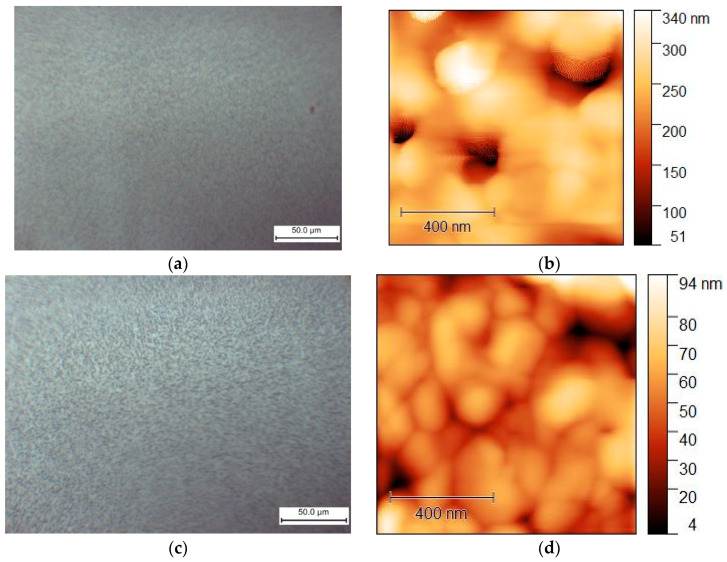
Morphology of films obtained from solutions in DMF:DMSO (1:4) obtained by centrifugation with an antisolvent (EA) for MAPbI_3_: (**a**) optical microscopy and (**b**) AFM (topography); and for MA_0.75_MEA_0.25_PbI_3_: (**c**) optical microscopy and (**d**) AFM (topography).

When using an antisolvent (ethyl acetate), the MAPbI_3_ and MA_0.75_MEA_0.25_PbI_3_ films also become continuous and homogeneous, as shown in Figure 10.

The MAPbI_3_ and MA_0.75_MEA_0.25_PbI_3_ films demonstrate comparable surface morphology and are free of dendritic structures. Therefore, MA_0.75_MEA_0.25_PbI_3_ films deposited using an antisolvent (EA) are suitable for perovskite solar cell fabrication. This is significant because an MEA cation fraction x < 0.25 does not significantly alter the absorption spectrum, as demonstrated in the previous results.

**Figure 11:**
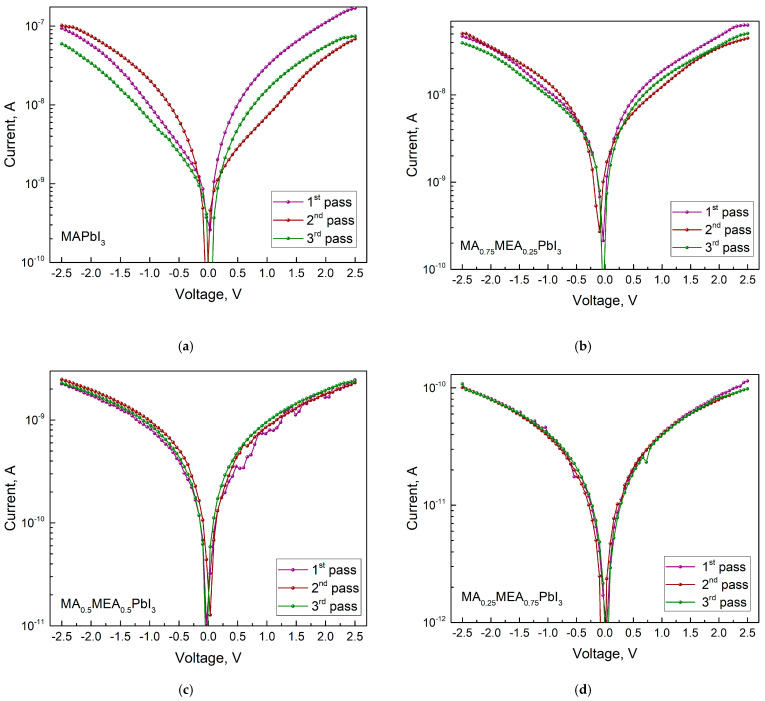
IVs in dark with three measurement passes for MA_x_MEA_1−x_PbI_3_: (**a**) MAPbI_3_, (**b**) MA_0.75_MEA_0.25_PbI_3_, (**c**) MA_0.5_MEA_0.5_PbI_3_, (**d**) MA_0.25_MEA_0.75_PbI_3_. The number of passes is indicated by a number in the figure.

The conductivity decreases as the MEA fraction (x) rises, as can be seen from the IV curves (Figure 11). Since the hybrid perovskite with a higher MEA proportion has a larger bandgap, this is an expected outcome. For MA_0.75_MEA_0.25_PbI_3_ the conductivity decreases insignificantly from the conductivity of MAPbI_3_, but a decrease in the IV hysteresis is observed.

**Figure 12:**
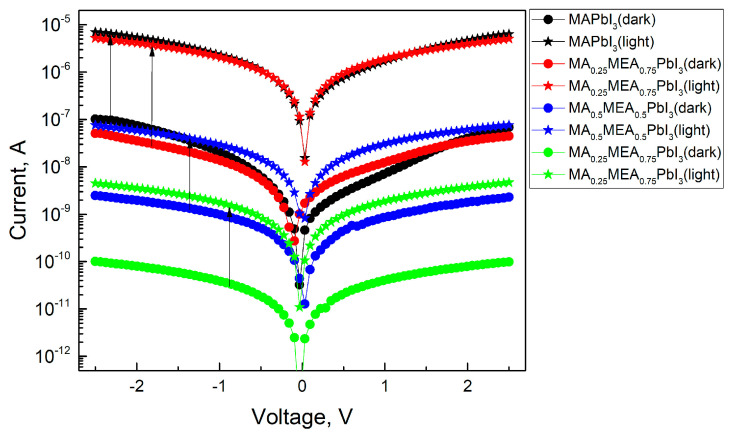
IVs of perovskite films in dark and under green LED irradiation.

As the MEA fraction increases, the hysteresis (difference in current values under forward and reverse voltage sweeps) decreases. The hysteresis in IV characteristics of hybrid perovskites arises from ion migration [71,72]. Thus, the reduced IV hysteresis in MA_x_MEA_1−x_PbI_3_ suggests improved stability in solar cells and photodetectors. IV measurements under green LED irradiation (Figure 12) reveal that all MA_x_MEA_1−x_PbI_3_ polycrystalline layers demonstrate strong photoresponse.

A slight decrease in the photoresponse with increasing MEA can be attributed to changes in the absorption spectrum of the samples. This trend would also be observed under irradiation with a solar radiation simulator. Nevertheless, the hybrid perovskite MA_x_MEA_1−x_PbI_3_ retains the strong photoresponse typical of hybrid perovskites, alongside a significant reduction in the hysteresis of the I–V characteristics.

## 4. Conclusions

In this study, hybrid 3D perovskites were synthesized using the monoethanolammonium cation. The resulting hybrid perovskites, MA_x_MEA_1−x_PbI_3_ (with MEA fraction up to 1 − x = 0.75), crystallize in a tetragonal perovskite structure. It was found that the MEAPbI_3_ compound does not form a stable perovskite phase without the addition of other cations.

An increase in the MEA fraction leads to a shift in the XRD peaks towards smaller 2Θ angles, indicating an expansion of the lattice parameters. Concurrently, changes in the bandgap and the shape of the absorption spectra were observed. For the hybrid perovskites MA_0.75_MEA_0.25_PbI_3_, MA_0.5_MEA_0.5_PbI_3_, MA_0.25_MEA_0.75_PbI_3_, the energy levels were determined. The experimental results suggest that these perovskites are compatible with widely used electron and hole transport layers. Hybrid perovskites MA_0.5_MEA_0.5_PbI_3_ and MA_0.25_MEA_0.75_PbI_3_ (and similar compositions) are promising materials for use in tandem solar cells. In particular, the bandgap of MA_0.5_MEA_0.5_PbI_3_ E_g_ ≈ 1.7 eV makes this hybrid perovskite optimal in terms of E_g_ value for use in tandem solar cells with Si.

Perovskites MA_0.5_MEA_0.5_PbI_3_ and MA_0.25_MEA_0.75_PbI_3_ form homogeneous, continuous polycrystalline layers when spin-coated from a DMF:DMSO (4:1) solution. Monoethanolammonium iodide in solution significantly affects the morphology of polycrystalline perovskite layers. The hybrid perovskite MA_x_MEA_1−x_PbI_3_ exhibits minimal hysteresis in the I–V characteristics, which indicates reduced ion migration. This suggests improved stability for devices such as solar cells, photodetectors, and X-ray detectors.

For a small fraction of MEA (x ≤ 0.25), changes in the crystal structure, absorption spectrum, and bandgap are minimal. However, a reduction in I–V hysteresis is observed, suggesting lower degradation in solar cells without compromising solar absorption. This work provides foundational insights into the synthesis and properties of 3D hybrid perovskites incorporating the monoethanolammonium cation.

## Data Availability

Data are contained within the article.
